# Sex differences late after tetralogy of Fallot repair: a systematic review and meta-analysis

**DOI:** 10.1007/s12055-025-02127-y

**Published:** 2025-12-05

**Authors:** Charis Qing Ying Tan, Emma Gardiner, Alison Zhu, David Ray Andrews, Jelena Saundankar

**Affiliations:** 1 Department of Cardiac Surgery, Perth Children’s Hospital, 15 Hospital Ave, Nedlands, WA 6009 Australia; 2 Department of Cardiology, Perth Children’s Hospital, 15 Hospital Ave, Nedlands, WA 6009 Australia; 3 Department of Cardiothoracic Surgery, Westmead Hospital, Cnr Hawkesbury Road and Darcy Rd, Westmead, NSW 2145 Australia

**Keywords:** Cardiac magnetic resonance, Right ventricular, Sex differences, Tetralogy of Fallot

## Abstract

**Purpose:**

Sex differences in long-term outcomes of patients with repaired tetralogy of Fallot (TOF) are increasingly recognised. However, the sex-specific timing of re-intervention is still unclear.

**Methods:**

A systematic review and meta-analysis was performed across 8 electronic databases from inception to February 2025. Inclusion criteria were studies that reported outcomes of sex differences in right ventricular (RV) volume, function, and right ventricular outflow tract obstruction (RVOTO) in patients after repaired TOF. Studies were identified and data were extracted by two independent reviewers. Data were extracted and pooled using random effects models and Review Manager 5.4 software.

**Results:**

The findings of three studies with 767 patients who underwent cardiac magnetic resonance (CMR) and one study of 148 patients who underwent echocardiography were included. The mean QRS interval reported in three studies was 148.9 ± 27.9 ms in males and 134.7 ± 24.6 ms in females; this was significant in all three studies [1–3]. Males had higher indexed right ventricular end-diastolic volume (RVEDVi) and indexed right ventricular end-systolic volume (RVESVi). In contrast, they had lower mean right ventricular ejection fraction (RVEF). Males had higher mean indexed left ventricular end-diastolic volume (LVEDVi) and mean indexed left ventricular end-systolic volume (LVESVi). Similarly, males had lower mean left ventricular ejection fraction (LVEF). Echocardiography findings showed that LVEF and strain were lower in males. Cardiopulmonary exercise test (CPET) findings reported in one study showed that males had a higher peak oxygen uptake and lower ventilation per unit of carbon dioxide production (VE/VCO_2_) slope than females.

**Conclusion:**

In view of significant long-term outcome differences in RV function and volume between males and females, we recommend a sex-specific threshold rather than a unisex threshold when it comes to consideration of the timing of re-intervention. More multi-centred studies are required to understand this better.

Central message

We have shown that there are long-term outcome differences between males and females. Therefore, we recommend sex-specific thresholds when it comes to consideration of timing for re-intervention.

**Table:** 

Aspect	Male	Female
ECG		
QRS length, ms	Longer	Shorter
Cardiac magnetic resonance imaging (MRI)		
RV Volume (ml/m2)	Larger	Smaller
LV Volume (ml/m2)	Larger	Smaller
RV Function (%)	Lower	Higher
LV Function (%)	Lower	Higher
Echocardiogram		
LV Volume (ml/m2)	Larger	Smaller
LV Function (%)	Lower	Higher
RV Strain (%)	Lower	Higher
LV Strain (%)	Lower	Higher
Exercise testing		
Peak oxygen uptake(ml/kg/minute)	Higher	Lower
VE/VCO2 Slope	Lower	Higher

Nb: LV = Left Ventricle, PR = Pulmonary Regurgitation, RV = Right Ventricle, RVEF = Right Ventricular Ejection Fraction, VE/VCO_2_= Ventilation per Unit of Carbon Dioxide Production

## Introduction

Tetralogy of Fallot (TOF) is the 5th most common congenital heart disease and the most common cyanotic congenital heart condition. With advancements in medical and surgical treatments, patients with repaired TOF are surviving far into adulthood. However, with progressive right ventricular (RV) dilatation and dysfunction, decision-making regarding the timing and threshold of re-intervention, such as pulmonary valve replacement (PVR), is still unclear [1–9]. Numerous studies have shown sex differences in right ventricular (RV) and left ventricular (LV) size and function found on cardiac magnetic resonance (CMR) and are currently included as standard practice in morphologically normal hearts [1, 10, 11].

In adults with repaired congenital heart disease, women have been shown to have a higher risk of pulmonary hypertension, a lower risk of endocarditis, and a lower risk of implantable cardioverter defibrillator (ICD) insertion and arrhythmia [12]. A growing number of publications have described sex differences long-term after TOF repair, such as RV remodelling, with men having lower right ventricular ejection fraction (RVEF).

However, current guidelines provide unisex thresholds for re-intervention. Therefore, we propose a separate sex cut-off for re-intervention after repair that has not been shown before [1–4, 12–17].

## Methods

**Fig. 1:**
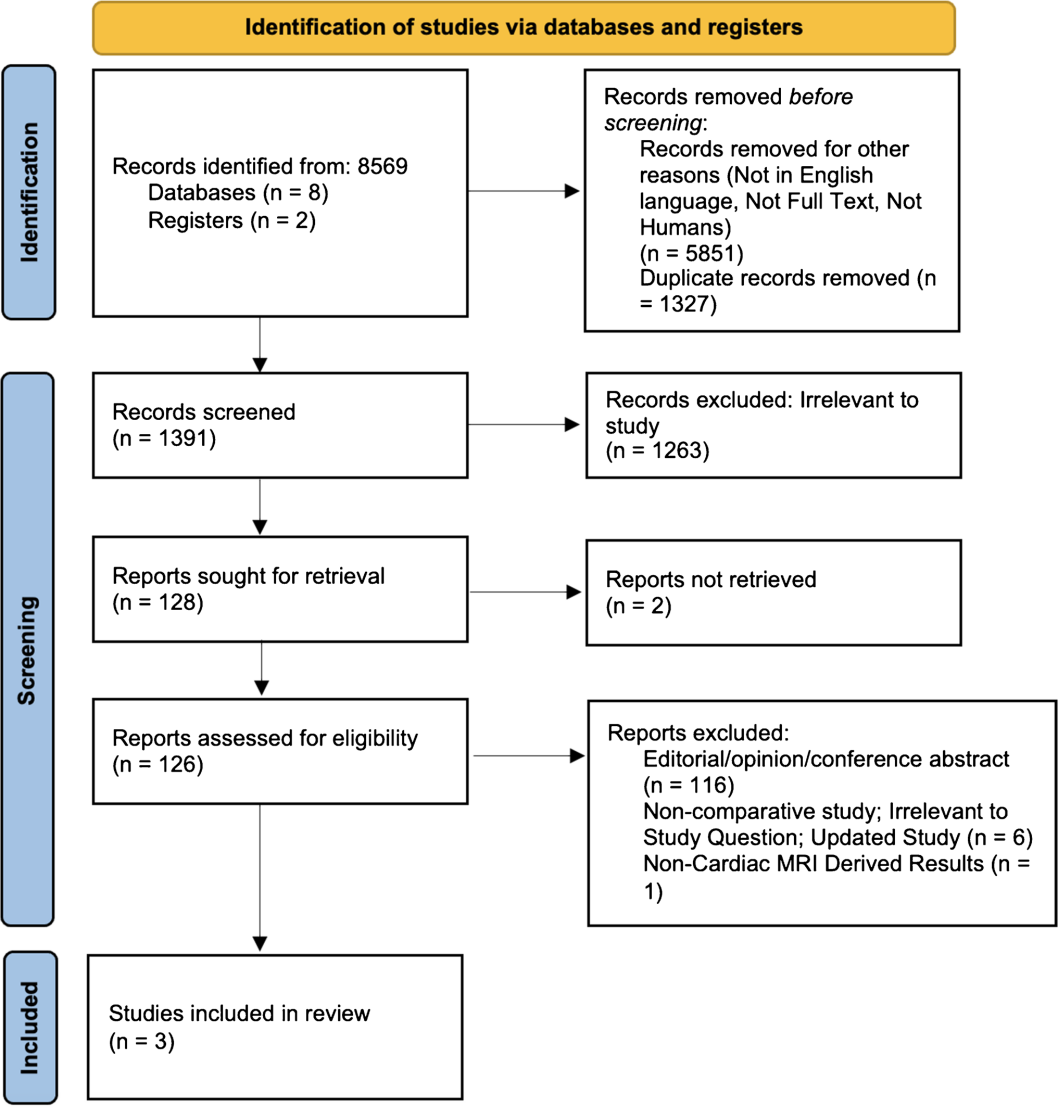
PRISMA table

This meta-analysis was performed in accordance with the Preferred Reporting Items for Systematic Reviews and Meta-Analyses (PRISMA) recommendations and guidelines (Fig. 1). The search strategy queried eight electronic databases including EMBASE, Ovid Medline, Scopus, the entire Cochrane Central Register of Controlled Trials (CCRCT), Cochrane Database of Systematic Reviews (CDSR), and the Database of Abstracts of Reviews of Effects (DARE) from inception to 03 Feb 2025. The search terms were “((tetralogy of Fallot OR TOF OR Fallot’s Tetralogy) OR (RVOTO OR right ventricular outflow tract obstruction adj3 surg)) AND ((Sex adj3 OR Gender adj3) AND (Diff* OR differences adj3))”. The references of previous systematic reviews were assessed to ensure no additional publications were missed.

### Selection criteria

Eligibility for inclusion in this systematic review included comparative studies that assessed the sex difference in right ventricular outflow tract obstruction (RVOTO), pulmonary regurgitation (PR), bi-ventricular volumes, and function in patients after TOF repair from echocardiograms or CMR. The exclusion criteria are: non-humans, non-English language papers, abstracts, expert opinion papers, non-comparative studies, and full papers that could not be retrieved. If there was an updated paper, the more recent one was included.

### Data extraction

For the assessed papers, data was extracted from the reviewed text, tables, and figures. All articles were independently reviewed, and data was extracted by two authors (CT and EG). Any discrepancies were resolved through discussion and inclusion of a third reviewer (AZ) until consensus was reached. The reference list of each included article was reviewed to further identify any other relevant studies.

### Outcomes of interest

Primary outcomes of interest were sex differences in indexed right ventricular end-systolic volume (RVESVi), indexed right ventricular end-diastolic volume (RVEDVi), PR or pulmonary regurgitation fraction, RVOTO, RVEF, indexed left ventricular end-systolic volume (LVESVi), indexed left ventricular end-diastolic volume (LVEDVi), left ventricular ejection fraction (LVEF), and cardiac index. Secondary outcomes included sex differences in ventricular arrhythmias (VA), implantable cardioverter defibrillator (ICD) rates, stroke rates, reoperation rates, and mortality.

The recorded parameters included were number of cases in the series, gender, age at surgery, height and weight at the time of index surgery and at enrolment, body surface area (BSA), body mass index (BMI), right ventricular outflow tract (RVOT) gradient, pulmonary regurgitant (PR) fraction, PR gradient, RVEDVi, RVESVi, RVEF, LVEDVi, indexed LVESVi, LVEF, and cardiac index.

### Statistical analysis

For continuous data with central tendency described using median values, the mean and standard deviation were estimated using calculations described by Wan and colleagues [18]. Data were presented as numbers and percentages or median and interquartile range (IQR).

Categorical and continuous variables were pooled using meta-analysis of proportions or means, as appropriate, using a random effects model. Review Manager v5.4.1 (Cochrane Collaboration, Copenhagen, Denmark) was used for performing pooled analysis for the selected outcomes. Due to the varied patient populations, a random effects model was chosen for all analyses. Estimated effect measures were performed for binary outcomes using the Mantel-Haenszel statistical method.

The odds ratio (OR) with a 95% confidence interval (CI) calculated in this study was based on the odds of an event for females compared to males. Outcomes were only pooled if they were reported by all three studies. For outcome data, data significance and heterogeneity were assessed using the Cochrane *Q* statistic and the *I*^2^ test statistic, respectively, with significance set at a *p* value < 0.05 and significant heterogeneity denoted by an *I*^2^ value > 50%. Thresholds for *I*^2^ values were considered as low, moderate, and high heterogeneity as 0–49%, 50–74%, and ≥ 75%, respectively.

## Results

### Patient characteristics

Three studies and 767 patients who underwent a cardiac MRI were included for meta-analysis [1, 2, 14]. There was one study (148 patients) that had echocardiography-reported outcomes [3]. Of the three included studies for meta-analysis, 412 (55%) were males and 355 (45%) were females. The mean weight at initial TOF repair was 17.9 ± 10.4 kg for males and 19.9 ± 18.5 kg for females, and this was reported in two studies (Sarikouch et al., Hagdorn et al.; total 727 patients) [1, 2].

The mean weight at enrolment was 69.6 ± 22.4 kg for males and 56.0 ± 15.0 kg for females; this was reported in two studies (Sarikouch et al., Pettit et al.; *p* < 0.001; 0.002) [1, 14]. The mean height at enrolment was 170.8 ± 13.7 cm for males and 157.8 ± 10.6 cm for females. This was reported in two studies and was significant (Sarikouch et al., Pettit et al.; *p* = 0.001; 0.00003) [1, 14]. The mean BSA was reported in all three studies: 1.8 ± 0.35 in males and 1.54 ± 0.278 in females and was significant (*p* = 0.001; 0.001; 0.0002).

### Operative characteristics

**Fig. 2:**
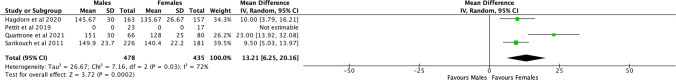
Forest plot of QRS interval

Type of initial TOF pathology was reported only in one study (Sarikouch et al.) and 365 (90%) patients had classical TOF and 42 (10%) had TOF with pulmonary atresia (PA) [1]. Where 309 (76%) patients had a primary repair and 80 (20%) patients had received a palliation procedure (modified Blalock-Taussig shunt) prior to their repair [1]. Two studies described the initial repair strategy where a transannular patch was used in 131 (18%) males and 125 (17%) females across 727 patients, and the remainder received trans atrial-transpulmonary annular sparing strategies [1, 2]. The mean QRS interval reported in three of four studies was 148.9 ± 27.9 ms in males and 134.7 ± 24.6 ms in females (*p* = 0.001; 0.003; 0.001) (Fig. 2) [1–3].

Only two studies reported maximum RVOT gradients [1, 2]. The mean peak RVOT gradient was 22.4 ± 15.3 in males and 19.8 ± 8.6 in females (*p* = 0.143; 0.83) [1, 2].

The studies included and results are described in Table 1 and Table 2 (CMR findings) and Table 3 (echocardiogram findings).

**Table 1:** Study characteristics

Author and year (country) (Reference)	Total no. (M/F)	Recruitment period	Method and follow-up	Imaging	Main findings
Sarikouch 2011 (Germany) [1]	407 (226/181)	2005–2008	Multi-centre, prospective cohort	CMR	No sex difference in peak RVOT gradients; but males had larger indexed RV volumes, lower RVEF, and longer QRS
Hagdorn 2020 (Netherlands: Stanford + Groningen) [2]	320 (163/157)	2007–2016	Multi-centre, retrospective cohort	CMR	No sex difference in peak RVOT gradients; but males showed larger RV/LV volumes and lower Bi-V EF
Pettit 2019 (USA) [14]	40 (23/17)	2011–2017	Single-centre, retrospective cohort	CMR	No sex difference in peak RVOT gradients, PR, and RVEF; males had larger indexed RV/LV volumes and lower LVEF
Quattrone 2021 (Norway) [3]	148 (68/80)	1970–2020	Single-centre, retrospective cohort	Echo	Males had lower LVEDVi and LVEF but higher LVESVi and RVOT diameter and higher QRS but no differences to incidence of VA and ICD insertion

Nb: *CMR*, cardiac magnetic resonance (imaging); *M*, males; *F*, females; *Bi-V*, bi-ventricle; *RVOT*, right ventricular outflow tract; *RV*, right ventricle; *RVEF*, right ventricular ejection fraction; *LV*, left ventricle; *LVEF*, left ventricular ejection fraction; *LVEDVi*, indexed left ventricular end-diastolic volume; *LVESVi*, indexed left ventricular end-systolic volume; *EF*, ejection fraction; *USA*, United States of America

**Table 2:** CMR results

Author	Imaging	Mean RVEDVi (ml/m^2^)	Mean RVESVi (ml/m^2^)	Mean LVEDVi (ml/m^2^)	Mean LVESVi (ml/m^2^)	Mean LVEF (%)
Sarikouch 2011 [1]	CMR	M, 124.8 ± 34.7 F, 114.9±29.9	M, 65.2 ± 25.7 F, 55.5 ± 20.1	M, 83.8 ± 18.5 F, 77.5 ± 14.7	M, 37.3 ± 14.4 F, 32.5 ± 9.8	M, 56.0 ± 8.4 F, 58.2 ± 8.3
Hagdorn 2020 [2]	CMR	M, 124.5 ± 38.4 F, 113.2 ± 27.5	M, 64.1 ± 23.4 F, 53.6 ± 18.8	M, 80.2 ± 18.7 F, 74.0 ± 16.5	M, 34.0 ± 12.6 F, 29.5 ± 8.4	M, 57.4 ± 7.85 F, 60.0 ± 7.48
Pettit 2019 [14]	CMR	M, 134.0 ± 35.3 F, 115.5 ± 30.4	M, 70.0 ± 22.2 F, 56.1 ± 20.2	M, 89.0 ± 17.2 F, 71.4 ± 13.9	M, 41.3 ± 9.8 F, 29.5 ± 8.1	M, 53.8 ± 5.5 F, 58.9 ± 6.0

Nb: *CMR*, cardiac magnetic resonance (imaging); *M*, males; *F*, females; *RV*, right ventricle; *RVEDVi*, indexed right ventricular end-diastolic volume; *RVESVi*, indexed right ventricular end-systolic volume; *LV*, left ventricle; *LVEF*, left ventricular ejection fraction; *LVEDVi*, indexed left ventricular end-diastolic volume; *LVESVi*, indexed left ventricular end-systolic volume

**Table 3:** Echocardiogram results

Author	Imaging	LV strain (%)	RV strain (%)	Mean LVEDVi (ml/m^2^)	Mean LVESVi (ml/m^2^)	Mean LVEF (%)
Quattrone 2021 [3]	Echocardiogram	M, − 15.8 ± 3.1 F, − 18.8 ± 3.2	M, − 19.1 ± 4.1 F, − 15.8 ± 3.9	M, 43.0 ± 12.0 F, 45.0 ± 13.0	M, 21.0 ± 7.0 F, 20.0 ± 6.0	M, 51.0 ± 8.0 F, 55.0 ± 8.0

Nb: *CMR*, cardiac magnetic resonance (imaging); *M*, males; *F*, females; *RV*, right ventricle; *LV*, left ventricle; *LVEF*, left ventricular ejection fraction; *LVEDVi*, indexed left ventricular end-diastolic volume; *LVESVi*, indexed left ventricular end-systolic volume

**Fig. 3:**
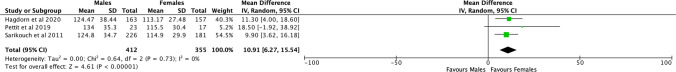
Forest plot of indexed right ventricular end-diastolic volume (RVEDVi) by CMR

**Fig. 4:**
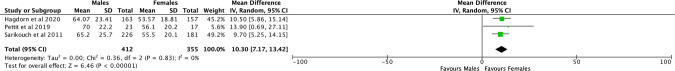
Forest plot of indexed right ventricular end-systolic volume (RVESVi) by CMR

**Fig. 5:**
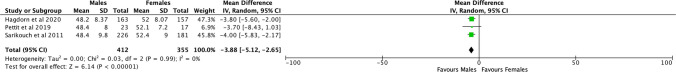
Forest plot of right ventricular ejection fraction (RVEF) by CMR

**Fig. 6:**
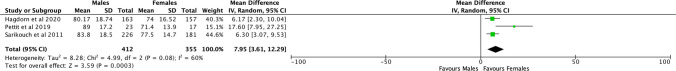
Forest plot of indexed left ventricular end-diastolic volume (LVEDVi) by CMR

**Fig. 7:**
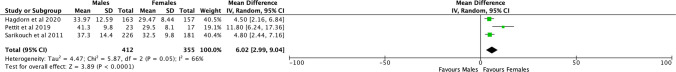
Forest plot of indexed left ventricular end-systolic volume (LVESVi) by CMR

**Fig. 8:**
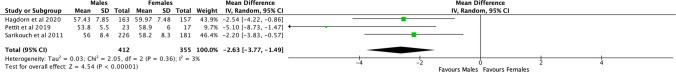
Forest plot of left ventricular ejection fraction (LVEF) by CMR

The sex differences in CMR variables were analysed in all three studies, and their pooled analysis and forest plots are as below and illustrated in Figs. 3, 4, 5, 6, 7, and 8 [1, 2, 14].

The mean RVEDVi was 127.8 ± 36.2 for males and 114.5 ± 29.3 for females in three studies, this was significant in two of the three studies, and significant in the pooled analysis (*p* = 0.003; 0.007, 0.12, *p* < 0.00001) (Fig. 3) [1, 2, 14]. The mean indexed RVESVi was 66.4 ± 23.8 for males and 55.1 ± 19.7 for females, this was significant for all three studies, and significant in the pooled analysis (*p* = 0.001; 0.001; 0.04, *p* < 0.00001) (Fig. 4) [1, 2, 14]. The mean RVEF was 48.3 ± 8.72 in males and 52.2 ± 8.09 in females. Although they were only significant in two of the three studies, they were significant in the pooled analysis (*p* = 0.019; 0.001; 0.1, *p* < 0.00001) (Fig. 5) [1, 2, 14].

The mean LVEDVi that reported in all three studies was 84.3 ± 18.1 in males and 74.3 ± 15.0 in females and was significant in the pooled analysis (*p* = 0.001; 0.002; 0.002, *p* < 0.0003) (Fig. 6). The mean indexed LVESVi was reported in all three studies and was 37.5 ± 12.3 in males and 30.5 ± 8.78 in females, this was statistically significant in all three studies and in the pooled analysis (*p* = 0.001; 0.001; 0.0006, *p* < 0.0001) (Fig. 7). The mean LVEF was reported in all three studies. This was 55.7 ± 7.25 in males and 59.0 ± 7.26 in females, and this was statistically significant (*p* = 0.012; 0.013; 0.02, *p* = 0.00001) (Fig. 8) [1–3, 14].

Quattrone et al. reported echocardiography findings in patients with repaired TOF. They found that males had lower LV and RV global longitudinal strain (*p* = 0.001; 0.001) than females and, therefore, impaired LV and RV function. They also found that males had longer QRS duration, although that did not impact patients having VA (*p* = 0.06). The incidence of VA was associated with higher right ventricular basal diameter (RVD1; *p* = 0.01), lower RV strain (*p* = 0.04), lower cardiac function (LVEF; RVEF, *p* = 0.02), and N-terminal pro-brain natriuretic peptide (NT-proBNP) over reference range (*p* < 0.001) [3].

Sarikouch et al. performed a follow-up study on the same population of patients looking at results from exercise testing after repaired TOF. They found that relative to sex-matched healthy controls, women with repaired TOF had larger RV end-systolic volumes, lower RV ejection fraction, lower RV muscle mass, higher ventilation per unit of carbon dioxide production (VE/VCO_2_) slopes, lower peak oxygen uptake, and lower peak heart rate [19].

A summary of the results of sex differences is described in Table 4.

**Table 4:** Summary of results

Aspect	Male	Female
ECG		
QRS length, ms	Longer	Shorter
CMR		
RV volume (ml/m^2^)	Larger	Smaller
LV volume (ml/m^2^)	Larger	Smaller
RV function (%)	Lower	Higher
LV function (%)	Lower	Higher
Echocardiogram		
LV volume (ml/m^2^)	Larger	Smaller
LV function (%)	Lower	Higher
RV strain (%)	Lower	Higher
LV strain (%)	Lower	Higher
Exercise testing		
Peak oxygen uptake (ml/kg/minute)	Higher	Lower
VE/VCO_2_ slope	Lower	Higher

Nb: *LV*, left ventricle; *PR*, pulmonary regurgitation; *RV*, right ventricle; *RVEF*, right ventricular ejection fraction; *VE/VCO*_*2*_, ventilation per unit of carbon dioxide production

### Mortality and morbidity

None of the four studies reported mortality or morbidity in terms of cerebrovascular accident [1–3, 14]. Only one study reported rates of VA (26% in males, 21% in females, *p* = 0.45) and implantable cardioverter defibrillator (ICD) insertions (19% in males, 11% in females, *p* = 0.18) [3].

## Discussion

### TOF and sex differences

TOF is the 5th most common congenital heart disease and the most common cyanotic congenital heart condition. With advancements in medical and surgical treatments, patients with TOF are surviving far into adulthood [1–7]. Numerous studies have noted sex differences in outcomes after congenital cardiac surgery in general as well as post TOF repair; however, current guidelines provide unisex thresholds for re-intervention post TOF repair [1–4, 12, 14–17]. This systematic review and meta-analysis is the first to analyse long-term sex differences in patients after repaired TOF. Based on our findings, we propose a separate sex cut-off for re-intervention after repaired TOF.

### Pulmonary valve replacement

It is understood that the progression of pulmonary regurgitation and a degree of RVOTO after the initial repair may be well tolerated without major clinical impact for decades. Ultimately, when pressure and volume overload exceed thresholds, their impact on LV and RV function is a major key predictor of morbidity and mortality [4, 5, 15, 20]. The European Society of Cardiology Guidelines in 2020, the American Heart Association Scientific Statement in 2024, and other studies have suggested class I recommendations for PVR in symptomatic patients and class 2a recommendations for PVR in asymptomatic patients with severe PR and/or RVOTO when RVESVi > 80 ml/m^2^ or RVEDVi > 160 ml/m^2^ or progression of tricuspid regurgitation to moderate or progressive RV systolic dysfunction or RVOTO with right ventricular systolic pressure (RVSP) > 80 mmHg [4, 6, 21]. PVR decreases the volume overload of the RV and, if timed well, will allow for RV remodelling and reversibility of RV dysfunction.

Fan et al. demonstrated the physiological impact of gender differences on RV dysfunction. They found that oestrogen promotes endogenous antioxidant enzymes and in turn, lowers rates of associated myocardial hypertrophy and fibrosis. Oestrogen also positively impacts myocardial energy metabolism as well as regulates calcium homeostasis [20].

Our findings have shown that the RVEDVi for both males and females at the time of enrolment in all four studies was generally lower than the guideline suggested thresholds; however, the RVESVi was higher. When we compared between both sexes, we found that males had higher volumes (RVEDVi, RVESVi, LVEDVi, LVESVi) but lower function (RVEF and LVEF) than females at the time of enrolment, and this was significant in the pooled analysis.

Previous studies have shown that females not only show normal RV function at the time of PVR, but they show a higher rate of reversibility of RVEDVi likely attributed to the protective effects of oestrogen in maintaining RV function and pulmonary vascular integrity [20].

### Arrhythmias and ICD

Arrhythmias and prolonged QRS occur at a rate of 24% after repaired TOF. [5, 15]. The 2020 ESC recommends ICD insertion when QRS > 180 ms, there is LV dysfunction, non-sustained ventricular tachycardia (NSVT), symptomatic ventricular tachycardia (VT), extensive RV scarring on CMR, and inducible VT at programmed electrical stimulation [21]. High RVEDVi has been shown to lead to prolonged QRS and VT, and therefore sudden cardiac death (SCD). Previous studies have noted that QRS duration decreases significantly post-PVR surgery [6, 22].

We have shown that the QRS duration is longer in males than females and this is significant. Although prolonged QRS duration was initially postulated to be the primary cause of sudden cardiac death (SCD) in this cohort of patients [5, 15, 23]. Quattrone et al showed that there were no differences in QRS duration in those who did and did not have ventricular arrhythmias (VA). They also found no sex differences between QRS duration and incidence of VA, and showed that although males (59%) had a higher rate of ICD insertion than females (41%) after repaired TOF, there were no differences in the incidence of ventricular arrhythmias (VA) [3].

Waldmann et al. found that women with TOF at high risk for SCD have a similar benefit/risk balance from ICD therapy compared with men [7].

With regard to risk factors for VA, Quattrone et al. have found that higher RV basal diameter, lower RV strain, lower ejection fraction, and NT-proBNP over the reference range are significant [3].

### Exercise testing

Sarikouch et al. performed a follow-up study comparing sex-specific differences between repaired TOF patients and sex-matched normal healthy subjects. They showed that when compared with sex-matched controls, RV systolic function and exercise capacity were lower in women than in men. Reduced peak oxygen uptake and higher VE/VCO_2_ slope are strong predictors of morbidity and mortality. Females are potentially at a higher risk of adverse outcomes when compared with their male counterparts [19].

### Heterogeneity

All four studies include patients with different types of initial TOF repair. Although this makes the pre-operative characteristics heterogenous, they have proven that there are no sex differences between their initial TOF repair strategies [1–3].

However, we encourage future studies to include a more homogenous population of patients with post-operative pulmonary regurgitation after an initial strategy of transannular patch for their index TOF repair, as this would allow for sex-specific RV volume thresholds to be more relevant in predicting timing of re-intervention.

## Limitations

The design of the four studies included patients in a cross-sectional period after repaired TOF. This created a heterogenous cohort, which included adults and children, patients with different initial TOF repair strategies (with and without transannular patch) as well as those with reoperation after their initial TOF repair. This may cause selection bias and confound our results, due to potential RV or LV remodelling post-redo surgery in some and no remodelling in others. We therefore propose future studies include only patients who had predominant longstanding pulmonary regurgitation after repaired TOF to allow for more accurate assessment of RV volumes and their implications.

Despite the extensive literature review, there were only four studies that compared the sex differences for patients after repaired TOF, of which three used data from CMR. Only two studies exclusively compared peak RVOT gradients post-operatively between both sexes and therefore a pooled meta-analysis could not be performed. As such, we could only compare LV function, bi-ventricular volume, and size differences as a threshold for re-intervention.

## Conclusion

We have shown that there are long-term outcome differences between males and females after repaired TOF on imaging and exercise testing. Therefore, we propose a sex-specific threshold rather than a unisex threshold when it comes to the timing of re-intervention in this cohort of patients. To achieve this, we suggest more multi-centred studies with a more homogenous cohort, such as patients with free pulmonary regurgitation after transannular patch repair. This is to better understand their long-term sex differences in bi-ventricular volumes and function and, therefore, allow us to better delineate the sex-specific thresholds for re-intervention more accurately. Lastly, we suggest further translational or molecular research into the sex differences in myocardial remodelling.

## Data Availability

All data is in public domain, no new data was generated.

## References

[1] Sarikouch S, Koerperich H, Dubowy KO, Boethig D, Boettler P, Mir TS, et al. Impact of gender and age on cardiovascular function late after repair of tetralogy of Fallot: percentiles based on cardiac magnetic resonance. Circ Cardiovasc Imaging [Internet]. 2011 [cited 2024 Oct 4];4:703–11. Available from: https://www.ahajournals.org/doi/10.1161/CIRCIMAGING.111.963637. 10.1161/CIRCIMAGING.111.963637 21908707

[2] Hagdorn QAJ, Beurskens NEG, Gorter TM, Eshuis G, Hillege HL, Lui GK. Sex differences in patients with repaired tetralogy of Fallot support a tailored approach for males and females: a cardiac magnetic resonance study. Int J Cardiovasc Imaging. 2020;36:1997–2005. 10.1007/s10554-020-01900-x. 32472300 10.1007/s10554-020-01900-x PMC7497497

[3] Quattrone A, Lie OH, Nestaas E, De Lange C, Try K, Lindberg HL, et al. Long-term follow-up and sex differences in adults operated for tetralogy of Fallot. Open Heart [Internet]. 2021 [cited 2024 Oct 4];8:e001738. Available from: https://openheart.bmj.com/lookup/doi/10.1136/openhrt-2021-001738. 10.1136/openhrt-2021-001738 PMC8524375 34663747

[4] Geva T, Wald RM, Bucholz E, Cnota JF, McElhinney DB, Mercer-Rosa LM, et al. Long-term management of right ventricular outflow tract dysfunction in repaired tetralogy of Fallot: a scientific statement from the American Heart Association. Circulation. 2024;150:e689–707. 10.1161/CIR.0000000000001291. 39569497 10.1161/CIR.0000000000001291

[5] Romeo JLR, Takkenberg JJM, Cuypers JAAE, De Groot NMS, Van De Woestijne P, Bruining N, et al. Timing of pulmonary valve replacement in patients with corrected Fallot to prevent QRS prolongation. Eur J Cardiothorac Surg [Internet]. 2020 [cited 2025 Apr 26];58:559–66. Available from: https://academic.oup.com/ejcts/article/58/3/559/5810148. 10.1093/ejcts/ezaa049 PMC7453033 32191321

[6] Pandya B, Quail MA, Cullen S. Clinical issues and outcomes in adults following repair of tetralogy of Fallot. Curr Treat Options Cardiovasc Med. 2013;15:602–14. 10.1007/s11936-013-0264-3. 23873585 10.1007/s11936-013-0264-3

[7] Waldmann V, Bouzeman A, Duthoit G, Koutbi L, Bessière F, Labombarda F, et al. Sex differences in outcomes of tetralogy of Fallot patients with implantable cardioverter-defibrillators. JACC Clin Electrophysiol [Internet]. 2022 [cited 2025 Apr 26];8:1304–14. Available from: https://linkinghub.elsevier.com/retrieve/pii/S2405500X22006776. 10.1016/j.jacep.2022.06.024 36266008

[8] Ammash NM, Dearani JA, Burkhart HM, Connolly HM. Pulmonary regurgitation after tetralogy of Fallot repair: clinical features, sequelae, and timing of pulmonary valve replacement. Congenit Heart Dis. 2007;2:386–403. 10.1111/j.1747-0803.2007.00131.x. 18377431 10.1111/j.1747-0803.2007.00131.x

[9] Hallbergson A, Gauvreau K, Powell AJ, Geva T. Right ventricular remodeling after pulmonary valve replacement: early gains, late losses. Ann Thorac Surg [Internet]. 2015 [cited 2021 Sept 4];99:660–6. Available from: https://linkinghub.elsevier.com/retrieve/pii/S0003497514018256. 10.1016/j.athoracsur.2014.09.015 25483002

[10] St. Pierre SR, Peirlinck M, Kuhl E. Sex matters: a comprehensive comparison of female and male hearts. Front Physiol [Internet]. 2022 [cited 2025 Aug 17];13:831179. Available from: https://www.frontiersin.org/articles/10.3389/fphys.2022.831179/full. 10.3389/fphys.2022.831179 PMC8980481 35392369

[11] Salton CJ, Chuang ML, O’Donnell CJ, Kupka MJ, Larson MG, Kissinger KV, et al. Gender differences and normal left ventricular anatomy in an adult population free of hypertension. J Am Coll Cardiol [Internet]. 2002 [cited 2025 Aug 17];39:1055–60. Available from: https://linkinghub.elsevier.com/retrieve/pii/S0735109702017126. 10.1016/s0735-1097(02)01712-6 11897450

[12] Verheugt CL, Uiterwaal CSPM, Van Der Velde ET, Meijboom FJ, Pieper PG, Vliegen HW, et al. Gender and outcome in adult congenital heart disease. Circulation [Internet]. 2008 [cited 2025 Apr 26];118:26–32. Available from: https://www.ahajournals.org/doi/10.1161/CIRCULATIONAHA.107.758086. 10.1161/CIRCULATIONAHA.107.758086 18559697

[13] Warnes CA. Sex differences in congenital heart disease: should a woman be more like a man? Circulation [Internet]. 2008 [cited 2025 Apr 26];118:3–5. Available from: https://www.ahajournals.org/doi/10.1161/CIRCULATIONAHA.108.785899. 10.1161/CIRCULATIONAHA.108.785899 18591448

[14] Pettit KA, Francois CJ, Aggarwal NR, Hess TM, Bartlett HL. Sex-specific differences in ventricular dimensions in repaired tetralogy of Fallot: a retrospective study. Pediatr Cardiol. 2019;40:1530–5. 10.1007/s00246-019-02181-5. 31401720 10.1007/s00246-019-02181-5

[15] Lee MGY, Yao JV, Binny S, Larobina M, Skillington P, Grigg LE, et al. Long-term outcome of adult survivors of tetralogy of Fallot. Int J Cardiol Congenit Heart Dis [Internet]. 2021 [cited 2024 Oct 4];4:100147. Available from: https://linkinghub.elsevier.com/retrieve/pii/S2666668521000719.

[16] Marelli A, Gauvreau K, Landzberg M, Jenkins K. Sex differences in mortality in children undergoing congenital heart disease surgery: a United States population–based study. Circulation [Internet] 2010 [cited 2025 Apr 26];122(11_suppl_1). Available from: https://www.ahajournals.org/doi/10.1161/CIRCULATIONAHA.109.928325. 10.1161/CIRCULATIONAHA.109.928325 20837919

[17] Freilinger S, Andonian C, Beckmann J, Ewert P, Kaemmerer H, Lang N, et al. Differences in the experiences and perceptions of men and women with congenital heart defects: a call for gender-sensitive, specialized, and integrative care. Int J Cardiol Congenit Heart Dis [Internet]. 2021 [cited 2024 Oct 4];4:100185. Available from: https://linkinghub.elsevier.com/retrieve/pii/S2666668521001099.

[18] Wan X, Wang W, Liu J, Tong T. Estimating the sample mean and standard deviation from the sample size, median, range and/or interquartile range. BMC Med Res Methodol [Internet]. 2014 [cited 2022 July 15];14:135. Available from: https://bmcmedresmethodol.biomedcentral.com/articles/10.1186/1471-2288-14-135. 10.1186/1471-2288-14-135 PMC4383202 25524443

[19] Sarikouch S, Boethig D, Peters B, Kropf S, Dubowy KO, Lange P, et al. Poorer right ventricular systolic function and exercise capacity in women after repair of tetralogy of Fallot: a sex comparison of standard deviation scores based on sex-specific reference values in healthy control subjects. Circ Cardiovasc Imaging [Internet]. 2013 [cited 2025 Aug 17];6:924–33. Available from: https://www.ahajournals.org/doi/10.1161/CIRCIMAGING.112.000195. 10.1161/CIRCIMAGING.112.000195 24132714

[20] Fan Q, Wang Y, An Q, Ling Y. Right ventricular dysfunction following surgical repair of tetralogy of Fallot: molecular pathways and therapeutic prospects. Biomed Pharmacother [Internet]. 2025 [cited 2025 May 4];184:117924. Available from: https://linkinghub.elsevier.com/retrieve/pii/S0753332225001180. 10.1016/j.biopha.2025.117924 39983432

[21] Baumgartner H, De Backer J, Babu-Narayan SV, Budts W, Chessa M, Diller GP, et al. 2020 ESC Guidelines for the management of adult congenital heart disease. Eur Heart J [Internet]. 2021 [cited 2021 Oct 2];42:563–645. Available from: https://academic.oup.com/eurheartj/article/42/6/563/5898606. 10.1093/eurheartj/ehaa554 32860028

[22] Oosterhof T, Vliegen HW, Meijboom FJ, Zwinderman AH, Bouma B, Mulder BJM. Long-term effect of pulmonary valve replacement on QRS duration in patients with corrected tetralogy of Fallot. Heart [Internet]. 2007 [cited 2025 Apr 26];93:506–9. Available from: https://heart.bmj.com/lookup/doi/10.1136/hrt.2006.094169. 10.1136/hrt.2006.094169 PMC1861506 17065183

[23] Gatzoulis MA, Balaji S, Webber SA, Siu SC, Hokanson JS, Poile C, et al. Risk factors for arrhythmia and sudden cardiac death late after repair of tetralogy of Fallot: a multicentre study. The Lancet [Internet]. 2000 [cited 2024 Oct 4];356:975–81. Available from: https://linkinghub.elsevier.com/retrieve/pii/S0140673600027148. 10.1016/S0140-6736(00)02714-8 11041398

