# Correction to: Sex differences late after tetralogy of Fallot repair: a systematic review and meta‑analysis

**DOI:** 10.1007/s12055-026-02189-6

**Published:** 2026-02-26

**Authors:** Charis Qing Ying Tan, Emma Gardiner, Alison Zhu, David Ray Andrews, Jelena Saundankar

**Affiliations:** 1 Department of Cardiac Surgery, Perth Children’s Hospital, 15 Hospital Ave, Nedlands, WA 6009 Australia; 2 Department of Cardiology, Perth Children’s Hospital, 15 Hospital Ave, Nedlands, WA 6009 Australia; 3 Department of Cardiothoracic Surgery, Westmead Hospital, Cnr Hawkesbury Road and Darcy Rd, Westmead, NSW 2145 Australia

**Correction to: Indian Journal of Thoracic and Cardiovascular Surgery (January 2025) 42(1):113–122**

10.1007/s12055-025-02127-y

1. During the typesetting of the published article “Sex differences late after tetralogy of Fallot repair: a systematic review and meta‑analysis,” there were incorrect transcription of the data in the “**Abstract**” section (“Methods”). The incorrect and correct information is shown in this correction article. This does not affect the study's PRISMA table, data analysis, results, statistical significance, or the conclusions. A total of 3 studies were included for majority of the meta-analysis, and the figures and table remain accurate as published.

**Incorrect:**

A systematic review and meta-analysis was performed across 6 electronic databases from inception to February 2025.

**Correct:**

A systematic review and meta-analysis was performed across 8 electronic databases from inception to February 2025.

2- During the typesetting of the published article “Sex differences late after tetralogy of Fallot repair: a systematic review and meta‑analysis,” there were incorrect transcription of the information in the “**Discussion**” section (“Arrhythmias & Implantable Cardioverter Defibrillator (ICD)”). The incorrect and correct information is shown in this correction article.

**Published:**

We have shown that the QRS duration is longer in males than females and this is significant. Although prolonged QRS duration was initially postulated to be the primary cause of sudden cardiac death (SCD) in this cohort of patients [5,15,23].

**Should read:**

We have shown that the QRS duration is longer in males than females and this is significant. Although prolonged QRS duration was initially postulated to be the primary cause of sudden cardiac death (SCD) in this cohort of patients [5,15,23]. Quattrone et al showed that there were no differences in QRS duration in those who did and did not have ventricular arrhythmias (VA). They also found no sex differences between QRS duration and incidence of VA, and showed that although males (59%) had a higher rate of ICD insertion than females (41%) after repaired TOF, there were no differences in the incidence of ventricular arrhythmias (VA) [3].

3- During the typesetting of the published article “Sex differences late after tetralogy of Fallot repair: a systematic review and meta‑analysis,” there were incorrect transcription of the data in the **“Table 1: Study Characteristics”** section (“Author & Year (Country) (Reference)”). The incorrect and correct information is shown in this correction article. This does not affect the study's data analysis, results, statistical significance, or the conclusions.

**Incorrect:**

**Table:** 

**Author & Year (Country)** **(Reference)**	**Total No. (M/F)**	**Recruitment Period**	**Method & Follow-up**	**Imaging**	**Main findings**
Quattrone 2021 (Norway) [3]	148 (138/118)	1970-2020	Single Centre, Retrospective cohort	Echo	Males had lower LVEDVi and LVEF but higher LVESVi and RVOT diameter and higher QRS but no differences to incidence of VA and ICD insertion

**Correct:**

**Table:** 

**Author & Year (Country)** **(Reference)**	**Total No. (M/F)**	**Recruitment Period**	**Method & Follow-up**	**Imaging**	**Main findings**
Quattrone 2021 (Norway) [3]	148 (68/80)	1970-2020	Single Centre, Retrospective cohort	Echo	Males had lower LVEDVi and LVEF but higher LVESVi and RVOT diameter and higher QRS but no differences to incidence of VA and ICD insertion

4- During the typesetting of the published article “Sex differences late after tetralogy of Fallot repair: a systematic review and meta‑analysis,” there were incorrect transcription of the data in the **“Table 2.1: CMR Results”** section (“Author”). The incorrect and correct information is shown in this correction article. This does not affect the study's data analysis, results, statistical significance, or the conclusions.

**Incorrect:**

**Table:** 

Author	Imaging	Mean RVEDVi (ml/m^2^)	Mean RVESVi (ml/m^2^)	Mean LVEDVi (ml/m^2^)	Mean LVESVi (ml/m^2^)	Mean LVEF (%)
Sarikouch 2011 [1]	CMR	M:124.8±34.7 F: 14.9±29.9	M:65.2±25.7 F:55.5±20.1	M:83.8±18.5 F:77.5±14.7	M:37.3±14.4 F:32.5±9.8	M:56.0±8.4 F:58.2±8.3

**Correct:**

**Table:** 

Author	Imaging	Mean RVEDVi (ml/m^2^)	Mean RVESVi (ml/m^2^)	Mean LVEDVi (ml/m^2^)	Mean LVESVi (ml/m^2^)	Mean LVEF (%)
Sarikouch 2011 [1]	CMR	M:124.8±34.7 F: 114.9±29.9	M:65.2±25.7 F:55.5±20.1	M:83.8±18.5 F:77.5±14.7	M:37.3±14.4 F:32.5±9.8	M:56.0±8.4 F:58.2±8.3

